# Supplementary material for: AffyMAPSDetector: a software tool to characterize Affymetrix GeneChip™ expression arrays with respect to SNPs
Source: BMC Bioinformatics. 2007 Jul 30;8:276. doi: 10.1186/1471-2105-8-276 (PMC1959249; doi:10.1186/1471-2105-8-276)
Supplement: Additional file 13 — AffyMAPSDetector v1 source code. AffyMAPSDetector source code. [file 1471-2105-8-276-S13.zip › AffyMAPSDetector_v1_src/doc/jwf/class-use/WizardPanel.html]

Uses of Class jwf.WizardPanel


|  |  |  |  |
| --- | --- | --- | --- |
| **Overview** \| **Package** \| **Class** \| **Use** \| **Tree** \| **Deprecated** \| **Index** \| **Help** | | |  |
| PREV   NEXT | **FRAMES**    **NO FRAMES**     **All Classes** |  |  |


---


## **Uses of Class jwf.WizardPanel**

|  |  |
| --- | --- |
| Packages that use WizardPanel | |
| **affychip.snpdetector.ui** |  |
| **jwf** | This package contains classes that can be used to create and run a Wizard. |

Uses of WizardPanel in affychip.snpdetector.ui

|  |  |
| --- | --- |
| Subclasses of WizardPanel in affychip.snpdetector.ui | |
| `class` | `StepOnePanel`             Title: Probe SNP Detector |
| `class` | `StepThreePanel`             Title: Probe SNP Detector |
| `class` | `StepTwoPanel`             Title: Probe SNP Detector |

|  |  |
| --- | --- |
| Methods in affychip.snpdetector.ui that return WizardPanel | |
| `WizardPanel` | `StepTwoPanel.next()` |
| `WizardPanel` | `StepThreePanel.next()` |
| `WizardPanel` | `StepOnePanel.next()`             Get the next panel to go to. |

Uses of WizardPanel in jwf

|  |  |
| --- | --- |
| Subclasses of WizardPanel in jwf | |
| `class` | `NullWizardPanel`             An implementation of the base class used for implementing a panel that is displayed in a Wizard. |

|  |  |
| --- | --- |
| Methods in jwf that return WizardPanel | |
| `abstract  WizardPanel` | `WizardPanel.next()`             Get the next panel to go to. |
| `WizardPanel` | `NullWizardPanel.next()`             Get the next panel to go to. |

|  |  |
| --- | --- |
| Methods in jwf with parameters of type WizardPanel | |
| `void` | `Wizard.start(WizardPanel wp)`             Start this wizard with this panel. |

---


|  |  |  |  |
| --- | --- | --- | --- |
| **Overview** \| **Package** \| **Class** \| **Use** \| **Tree** \| **Deprecated** \| **Index** \| **Help** | | |  |
| PREV   NEXT | **FRAMES**    **NO FRAMES**     **All Classes** |  |  |


---
